# Chemotherapy in Cutaneous Melanoma: Is There Still a Role?

**DOI:** 10.1007/s11912-023-01385-6

**Published:** 2023-03-29

**Authors:** James P. Pham, Anthony M. Joshua, Ines P. da Silva, Reinhard Dummer, Simone M. Goldinger

**Affiliations:** 1 Medical Oncology, The Kinghorn Cancer Centre, St. Vincent’s Hospital Sydney, Darlinghurst, NSW Australia; 2 School of Clinical Medicine, UNSW Medicine and Health, St Vincent’s Hospital, Darlinghurst, NSW Australia; 3 Melanoma Institute Australia, The University of Sydney, Wollstonecraft, NSW Australia; 4 Medical Oncology, Blacktown Hospital, Blacktown, NSW Australia; 5 Department of Dermatology, University Hospital Zurich, Rämistrasse 100, 8091 Zurich, Switzerland; 6 Faculty of Medicine, University of Zurich, Zurich, Switzerland

**Keywords:** Melanoma, Chemotherapy, Immune checkpoint inhibitor, Targeted therapy

## Abstract

**Purpose of Review:**

In the preceding decade, the management of metastatic cutaneous melanoma has been revolutionised with the development of highly effective therapies including immune checkpoint inhibitors (specifically CTLA-4 and PD-1 inhibitors) and targeted therapies (BRAF and MEK inhibitors). The role of chemotherapy in the contemporary management of melanoma is undefined.

**Recent Findings:**

Extended analyses highlight substantially improved 5-year survival rates of approximately 50% in patients with metastatic melanoma treated with first-line therapies. However, most patients will progress on these first-line treatments. Sequencing of chemotherapy following failure of targeted and immunotherapies is associated with low objective response rates and short progression-free survival, and thus, meaningful benefits to patients are minimal.

**Summary:**

Chemotherapy has limited utility in the contemporary management of cutaneous melanoma (with a few exceptions, discussed herein) and should not be the standard treatment sequence following failure of first-line therapies. Instead, enrolment onto clinical trials should be standard-of-care in these patients.

## Introduction

The treatment of metastatic cutaneous melanoma has been revolutionised in the last decade with the introduction of immune-activating and targeted therapies. Prior to their development, chemotherapy was the mainstay of melanoma treatment — with 5-year survival rates of approximately 6–10% in patients with stage IV disease [1].

Treatment with immunotherapy, specifically immune checkpoint inhibition (ICI), has significantly improved melanoma survival compared to the chemotherapy era — with 5-year survival rates for single-agent PD-1 inhibitors of 34–44% in stage IV disease [2, 3] and 52% when combined with the CTLA-4 inhibitor ipilimumab [3]. As such, the PD-1 inhibitors nivolumab or pembrolizumab, alone or in combination with ipilimumab, are recommended as first-line systemic treatment for metastatic melanoma by the American Society of Clinical Oncology [4], the National Comprehensive Cancer Network [5] and the European Society of Medical Oncology [6].

Approximately, 70% of melanoma have activating mutations in the MAPK pathway, two thirds of which in the BRAF gene and are thus targetable by BRAF inhibitors (BRAFi) including vemurafenib, dabrafenib and encorafenib. When used in combination with MEK inhibitors (MEKi) (cobimetinib, trametinib or binimetinib), which target downstream kinases in the MAPK pathway, 5-year survival for BRAF inhibition ranges from 31 to 35% [7–9]. Thus, guidelines [4–6] also propose MAPK-targeted therapy (TT) as an option for BRAF-mutant patients.

No guidelines explore the role of cytotoxic chemotherapy in the contemporary management of melanoma. Therefore, it is relevant to examine whether this prototypical anticancer therapy still has a role in the management of patients with metastatic cutaneous melanoma and in what specific contexts.

## Chemotherapy in Melanoma

Table 1 summarises historical chemotherapy agents used for metastatic cutaneous melanoma, including clinical efficacy outcomes and toxicities.

**Table 1:** Summary of historical chemotherapy agents used for metastatic cutaneous melanoma, including clinical efficacy outcomes and toxicities

Chemotherapy	Objective response rate	Median overall survival	Notable common adverse events (frequency)
Alkylating agents			
Dacarbazine [10, 11]	9–29%	7–7.5 months	• Myelosuppression (45%) • Photosensitivity (10%) • Nausea (9%)
Temozolamide [12, 13]	9–29%	4.0–13.1 months	• Nausea (52%) • Pain (34%) • Myelosuppression (34%)
Fotemustine [14, 15]	12–47%	4.5–7.3 months	• Thrombocytopenia (94%) • Neutropenia (71%) • Nausea (20%)
Taxanes			
Paclitaxel [16–21]	0–36%	7–7.8 months	• Fatigue (50%) • Nausea (43%) • Anaemia (21%)
Nab-paclitaxel [22]	12–15%	9.6–12.6 months	• Neutropenia (46%) • Anaemia (23%) • Peripheral neuropathy (41%) • Fatigue (26%)
DHA-paclitaxel [23]	5.2–11%	8.9	• Neutropenia (61%) • Lymphopenia (3.1%) • Nausea (2.1%)
Docetaxel [24–26]	5.7–17%	6.5–13 months	• Neutropenia (83–95%) • Anaemia (70–83%) • Nausea (33–42%) • Peripheral oedema (19–60%)
Platinum agents			
Cisplatin [27, 28]	16.3–19.8%	7.6 months	• Peripheral neuropathy (93%) • Ototoxicity (24%) • Nephrotoxicity (19%)
Carboplatin [29]	19.2%	Not reported	• Thrombocytopenia (100%) • Nausea (58%) • Malaise (7.7%)
Carboplatin and cisplatin [30]	26.4%	12.5 months	• Nausea (93%) • Anaemia (93%) • Thrombocytopenia grade 4 (27%) • Leukopenia grade 4 (13%)
Combination chemotherapy protocols			
Carboplatin + paclitaxel [31–33]	9.6–20%	4.6–11.3 months	• Neutropenia (49.1%) • Peripheral neuropathy (14.9%) • Fatigue (14.1%)
Cisplatin + carmustine + dacarbazine + / − tamoxifen (*Dartmouth*) [34]	21–30%	7.7 months	• Neutropenia grade 3–4 (35%) • Thrombocytopenia grade 3–4 (57%) • Nausea grade 3–4 (18%)
Cisplatin + dacarbazine + vinblastine [35]	9.6–40%	7.6–9 months	• Nausea (83%) • Diarrhoea (34%) • Febrile neutropenia (10%)
Bleomycin + vincristine + dacarbazine + lomustine + / − interferon-α [36, 37]	13–24%	7.5–9.8 months	• Neutropenia (84%) • Anaemia (59%) • Fever (72%) • Peripheral neuropathy (66%)

### Alkylating Agents

Dacarbazine (DTIC) was the first cytotoxic chemotherapeutic agent approved by the FDA (in 1975) for metastatic melanoma and prior to contemporary treatments was considered standard-of-care [38]. Dacarbazine is metabolised by the liver into the active metabolite MTIC, which forms DNA lesions such as O^6^-methylguanine resulting in double-stranded DNA breaks [39]. The overall response rate (ORR) to dacarbazine varies from 9 to 29% in the first-line (1L) setting; however, durable complete responses are rare (< 2%) [10]. A meta-analysis of 20 trials of dacarbazine identified a median OS of 7.4 months when used as 1L therapy [11]. Notably, no trials compare dacarbazine with best supportive care (BSC); and thus, meaningful benefits to patients are unclear — despite it having become the main comparator for trials in metastatic melanoma after having been proposed as such following a Cochrane review in 2000 [10].

Temozolomide is another alkylating agent given orally which is metabolised to MTIC. Contrary to dacarbazine, no prior hepatic conversion is required [40]. A systematic review of 26 studies of temozolomide in the 1L and beyond first-line (1L +) setting identified variable ORR from 9 to 29%, while median OS ranged from 4.0 to 13.1 months [12]. A phase III trial comparing temozolomide with dacarbazine demonstrated similar median OS (7.7 vs 6.4 months, respectively) and ORR (13.5% vs 12.1%, respectively) [13] as 1L therapy. Unlike dacarbazine, temozolomide is able to cross the blood–brain barrier and thus may have superior efficacy against central nervous system (CNS) metastases. One retrospective study of 122 patients with intracranial disease reported a 7% ORR (1L) [41], while the risk of CNS relapse was reduced by 77% with temozolomide versus dacarbazine in another retrospective study [42].

Fotemustine is a nitrosourea which, in addition to DNA and RNA alkylation leading to unstable target lesions such as O^6^-chlorethylguanine, also inhibits DNA repair enzymes such as DNA polymerase and nucleotidyltransferase [43]. A phase III study demonstrated superior ORR for fotemustine compared to dacarbazine (15.2% vs 6.8%, respectively, *P* = 0.043) in the 1L, although median OS was not significantly improved (7.3 months vs 6.4 months, respectively; *P* = 0.059) [14]. Due to its lipophilicity, fotemustine can penetrate the blood–brain barrier, with a retrospective cohort study of 36 patients with intracranial disease demonstrating an ORR of 25.0% [15].

### Taxanes

Taxanes inhibit mitosis by enhancing tubulin polymerisation to stabilise microtubules and halting the cell cycle. Three phase II studies of paclitaxel (at 250 mg/m^2^/24 h every 21 days) in metastatic melanoma demonstrated ORRs of 12–36%, while median OS was not reported in any [16–18]. However, due to the high frequency of neutropenia, shorter-/lower-dose infusions (at 80 mg/m^2^/1 h every 7 days) are more commonly given [44]. Three phase II studies (all 1L +) reported ORRs of 0–15.6% and median OS of 7–7.8 months in melanoma with this regimen [19–21].

However, pre-medication with steroids (e.g. dexamethasone) and H2-antihistamines (e.g. famotidine) is frequently required to reduce hypersensitivity reactions (manifesting as hypotension, bronchospasm and angioedema) which occur in approximately 11% of patients [45]. These reactions are theorised to be secondary to paclitaxel itself or the polyethoxylated castor oil solvent (Cremophor® EL). Consequently, nanoparticle albumin-bound paclitaxel (Nab-paclitaxel) was developed without Cremophor and does not require pre-medication against hypersensitivity reactions while additionally demonstrating superior antitumour activity in vitro [46]. Nab-paclitaxel has been studied in one randomised phase III trial against dacarbazine in metastatic melanoma (1L) — with comparable ORRs (15 vs 11%, respectively, *P* = 0.24) and median OS (12.6 vs 10.5 months, *P* = 0.27) [22].

Another derivative taxane is docosahexanoic acid–paclitaxel (DHA-paclitaxel), consisting of a fatty acid (DHA)-paclitaxel conjugate. Murine pharmacokinetic studies highlight sixfold increased tumoural concentration by DHA-paclitaxel compared with Cremophor-bound paclitaxel [47]. DHA-paclitaxel has been compared in one phase III trial against dacarbazine in melanoma patients in the 1L setting — with comparable ORRs (5.2 vs 5.5%, respectively) and median OS (8.9 vs 7.5 months) [23].

Docetaxel is another taxane with approximately twice the potency of microtubule stabilisation compared to paclitaxel in vitro [48]. Single-agent docetaxel has been studied in three phase II trials in metastatic melanoma (both 1L and 1L +) — yielding ORRs of 5.7–7%, including two durable complete responses [24–26]. Median OS was only reported in two studies, ranging from 6.5 to 13 months.

### Platinum Chemotherapy

Single-agent platinum-based chemotherapy (‘platin’) regimens have demonstrated some activity against metastatic melanoma. Platins impair DNA repair mechanisms via platinum ion cross-linkage with purine bases on the DNA, inducing DNA damage and apoptosis. Historical trials of cisplatin reported ORRs of 6–15% in metastatic melanoma in the 1L + setting; however, OS was not reported [27]. One phase II trial of cisplatin in 89 patients highlighted an ORR of 19.8% and median OS of 7.6 months, with efficacy and tolerability not significantly improved by the addition of amifostine (a myelo- and nephro-protective thiol derivative) [28]. Nephrotoxicity was unacceptably frequent in the cohort (19.2%) — with three life-threatening cases (3.4%) and one death as a result (1.1%).

Carboplatin is a newer generation platin characterised by increased half-life but decreased potency compared to cisplatin [49]. One phase II trial of 26 patients (1L +) observed partial responses in five patients (ORR 19.2%) with a median duration of response of 5 months; however, median OS was not provided [29]. Notably, only one case of renal impairment was reported (3.8%). This is consistent with the different safety profiles of carboplatin compared with cisplatin — with increased myelosuppression but decreased nephrotoxicity [49].

A phase II trial of cisplatin–carboplatin combination chemotherapy was conducted aiming to increase total platinum dosage while mitigating the different side effect profiles of the two agents. Fifteen patients who had progressed on dacarbazine were treated, yielding an ORR of 26.4% including two complete responses (13.3%), with a median OS of 12.5 months [30]. Unlike with high-dose single-agent cisplatin, renal impairment was uncommon (13%), with no cases severe or life-threatening.

### Combination Chemotherapy Regimens

Combining chemotherapy agents targeting different aspects of the cancer cell cycle is standard for many cancers to overcome tumour resistance to cytotoxicity and optimise varying adverse event (AE) profiles of individual drugs. As such, numerous combination chemotherapy combinations have been trialled in metastatic melanoma [33, 34, 35, 37, 50] with varying degrees of efficacy (key combinations featured in Table1). However, there is no evidence that such combinations confer improved survival compared to single-agent dacarbazine — with a Cochrane review and meta-analysis in 2018 determining an OS hazard ratio (HR) of 0.99 (95%CI 0.85–1.16) for polychemotherapy versus single-agent chemotherapy, at the cost of increased treatment toxicities (HR 1.97, 95%CI 1.44–2.71) [51]. Many of such combinations utilise a dacarbazine backbone; however, a further meta-analysis of 20 randomised trials found no significantly improved survival between dacarbazine alone and dacarbazine-based polychemotherapy (HR 1.16, 95%CR 0.87–1.54) [11].

Notably, the sole prospective study comparing chemotherapy with BSC in melanoma evaluates a non-randomised multicentre cohort treated with either cisplatin, vinblastine and dacarbazine (CVD) or BSC as 1L treatment [35]. Initially planned for randomisation this was altered to ‘patient preference’ after inadequate recruitment for the BSC arm. While ORR and median OS were superior in patients receiving CVD compared to BSC (9.6 vs 0% and 137 vs 229 days respectively) this was confounded by greater frequency of CNS disease and worse performance status of the BSC arm at baseline and thus definitive benefit of chemotherapy could not be concluded. Furthermore there were no differences in quality-of-life (QoL) scoring between treatment arms after 8 weeks despite improved ORR and OS.

## Chemotherapy Versus Contemporary Treatment Options

The approval of ipilimumab, the first ICI, soon followed by vemurafenib, the first TT, in 2011 heralded a paradigm shift in the treatment of melanoma [52]. These agents rapidly became standard-of-care replacing chemotherapy, with a trend towards improved survival [53•]. Relevant prospective randomised trials comparing such therapies versus chemotherapy are summarised in Table 2.

**Table 2:** Summary of prospective randomised trials comparing immune checkpoint inhibitors and BRAF/MEK inhibitors versus cytotoxic chemotherapy in the treatment of metastatic melanoma

Study	Cohort	Immune checkpoint inhibitor (size)		Chemotherapy comparator (size)		Objective response rate (*P* value)		Progression-free survival HR (*P* value)		Death HR (*P* value)
Immune checkpoint inhibitor										
CA184-024	Untreated metastatic melanoma, brain metastases excluded	Ipilimumab plus dacarbazine (250)		Dacarbazine (252)		15.2% vs10.3% (0.09*)	0.76 (0.006)			0.69 (< 0.001)
NCT00257205	Untreated unresectable or metastatic melanoma, brain metastases excluded	Tremelimumab (328)		Investigators’ choice chemotherapy (327)		10.7% vs 9.8% (0.62*)	0.88 (0.48*)			0.88 (0.13*)
KEYNOTE-002	Metastatic melanoma progressed on ipilimumab and BRAF/MEK inhibitors if BRAF-mutant, brain metastases excluded	Pembrolizumab (2 mg/kg) (180)		Investigators’ choice chemotherapy (179)		21% vs 4% (< 0.0001)	0.57 (< 0.0001)			0.86 (0.12*)
		Pembrolizumab (10 mg/kg) (181)				25% vs 4% (< 0.0001)	0.50 (< 0.0001)			0.74 (0.010*)
CheckMate 037	Metastatic melanoma progressed on ipilimumab and BRAF/MEK inhibitors if BRAF-mutant, brain metastases excluded	Nivolumab (272)		Investigators’ choice chemotherapy (133)		31.7% vs 10.6%	1.0 (*P* not reported)			0.95 (0.72*)
CheckMate 066	Untreated metastatic melanoma, brain metastases excluded	Nivolumab (210)		Dacarbazine (208)		42.8% vs 14.4% (< 0.001)	0.42 (< 0.001)			0.46 (< 0.001)
NIBIT-M2	Untreated melanoma with active, asymptomatic CNS metastases	Ipilimumab plus nivolumab (27)		Fotemustine (27)		44.4% vs 0%	NR			0.44 (0.017)
BRAF inhibitors										
BRIM-3	Untreated metastatic melanoma, BRAF^V600E/K^ mutant	Vemurafenib (337)	Dacarbazine (338)		57% vs 9%		0.38 (< 0.0001)		0.77 (0.0068)	
BREAK-3	Untreated metastatic or unresectable melanoma, BRAF^V600E/K^ mutant	Dabrafenib (187)	Dacarbazine (63)		50% vs 6% (*P* not reported)		0.30 (< 0.0001)		0.77 (*P* not reported)	
MEK inhibitors										
NCT00338130	Untreated metastatic melanoma, excluding unstable brain metastases	Selumetinib (104)	Temozolamide (96)		5.8% vs 9.4% (*P* not reported)		1.07 (0.70*)		1.35 (0.10*)	
NEMO	Unresectable advanced or metastatic NRAS-mutant melanoma, untreated or progressed on ICIs	Binimetinib (269)	Dacarbazine (133)		15 vs 9% (0.015)		0.62 (< 0.001)		1.00 (0.50*)	
NCT01693068	Unresectable advanced or metastatic NRAS-mutant melanoma, untreated	Pimasertib (130)	Dacarbazine (64)		27% vs 14% (0.05)		0.59 (0.002)		0.89 (*P* not reported)	
METRIC	Metastatic or unresectable melanoma, BRAF^V600E/K^ mutant (treatment non-naïve and brain metastases allowed)	Trametinib (214)	Investigators’ choice chemotherapy (108)		22% vs 8% (0.01)		0.45 (< 0.001)		0.54 (0.01)	

not meeting pre-specified cut-offs for statistical significance

### Chemotherapy Versus Immune Checkpoint Inhibition

One phase III randomised trial compared ipilimumab plus dacarbazine versus dacarbazine alone (1L), demonstrating similar ORR (15.2 vs 10.3%; *P* = 0.09) but improved median progression-free survival (PFS) (HR 0.76, *P* = 0.006) and OS (11.0 vs 8.9 months; HR 0.69, *P* < 0.001) [54]. This corresponded to superior 5-year survival rates in the ipilimumab plus dacarbazine group compared to dacarbazine alone (18.2% vs 8.8%, *P* = 0.002) [55]. However, 5-year survival of ipilimumab alone in other studies of untreated metastatic melanoma is approximately 26% [3], and thus, the value of added dacarbazine is unclear. AEs were slightly increased with the addition of ipilimumab compared to single-agent dacarbazine — with 98.8% vs 94.0% developing any grade events and 56.3% vs 27.5% for grade 3 or 4 events.

Tremelimumab, another CTLA-4 inhibitor, was compared against investigators’ choice chemotherapy (dacarbazine or temozolomide) in another randomised trial (1L) [56]. This showed no significant difference in ORR between the tremelimumab and chemotherapy cohorts (10.7 vs 9.8%, respectively; *P* = 0.09). While overall PFS was similar in both groups (6 months PFS rate 20.3 vs 18.1%; *P* = 0.48), for the patients who did respond to tremelimumab, their duration of response was significantly longer compared to chemotherapy responders (median 35.8 vs 13.7 months, *P* = 0.001). While this suggests CTLA-4 blockade provides sustained anticancer activity in a small subset of patients, no biomarkers to predict response were identified. Importantly, median OS was similar in both groups — 12.6 months in the tremelimumab arm and 10.7 in the chemotherapy arm (*P* = 0.13), suggesting chemotherapy is non-inferior to CTLA-4 blockade in unselected cohorts.

KEYNOTE-002 is a randomised trial comparing pembrolizumab with investigators’ choice chemotherapy (carboplatin–paclitaxel, paclitaxel, carboplatin, dacarbazine or temozolomide) in ipilimumab and BRAF/MEKi-refractory melanoma (1L +) [57]. Two doses of pembrolizumab (2 mg/kg and 10 mg/kg) were studied, both demonstrating ORRs superior to chemotherapy (21% and 25% vs 4%, respectively; *P* < 0.0001 for each). Moreover, of the patients who responded to pembrolizumab, the duration was more likely to be sustained at 3 years — 73% in the pembrolizumab 2 mg/kg arm and 74% in the pembrolizumab 10 mg/kg arm versus 13% in the chemotherapy arm [58]. Six-month PFS of 34% and 38% in the pembrolizumab 2 mg/kg and 10 mg/kg arms, respectively, was also significantly higher than with chemotherapy of 16% (*P* < 0.0001 for both). Although there were no significant differences in median OS (13.4/14.7 months for pembrolizumab 2 mg/kg and 10 mg/kg doses versus 11.0 months for chemotherapy), this was likely confounded by high crossover rates upon progression on chemotherapy (55%) [58]. Moreover, severe AEs were less frequent with pembrolizumab than chemotherapy (grade 3 10%/13% vs 20%; grade 4 0%/0% vs 6%) [57], and, importantly, health-related QoL was preserved to a greater degree for patients receiving pembrolizumab compared to chemotherapy (*P* = 0.01 for both doses of pembrolizumab compared with chemotherapy) [59].

Checkmate-037 compared nivolumab with investigator’s choice chemotherapy (dacarbazine or carboplatin–paclitaxel) in ipilimumab and BRAF/MEKi-refractory melanoma (1L +) [60]. Nivolumab resulted in superior ORR in this cohort (31.7% vs 10.6%) including increased duration of response; however, PFS and OS were not significantly improved (3.1 vs 3.4 months and 16 vs 14 months, respectively) [61•]. The study authors attributed this discrepancy to more patients with worse prognostic markers at baseline in the nivolumab cohort (including CNS metastases and elevated lactate dehydrogenase) and high treatment crossover rate in the chemotherapy group upon disease progression (41%).

Checkmate-066 compared nivolumab against dacarbazine in patients who had progressed on ipilimumab (1L +), highlighting significantly higher ORR (42.8% vs 14.4%, *P* < 0.001) [62]. Moreover, a landmark 5-year analysis demonstrated that only 2 patients (< 1%) experienced durable complete responses to dacarbazine, compared to 18% with nivolumab. This corresponded to significantly increased PFS and OS compared to dacarbazine, with 5-year PFS of 28% vs 3% and OS of 39% vs 17%, respectively. Importantly, nivolumab was also shown to maintain health-related QoL in these patients, unlike dacarbazine [63].

Fotemustine was compared with ipilimumab plus fotemustine and ipilimumab plus nivolumab in patients with asymptomatic brain metastasis (NIBIT-M2) in the 1L setting, showing vastly inferior response rates in both fotemustine-containing arms compared to ipilimumab–nivolumab (0% and 19.2% vs 44.4%, respectively) [64••]. In addition, OS at 4 years was significantly higher in the ipilimumab–nivolumab arm than the fotemustine and fotemustine–ipilimumab arms (41% vs 10.9% vs 10.3%, respectively).

### Chemotherapy Versus Targeted Therapy

BRIM-3 was the randomised pivotal trial evaluating vemurafenib against dacarbazine in BRAF-mutant metastatic melanoma (1L), showing significant increased ORR (57% vs 9%, respectively; *P* < 0.001) and PFS (6.9 vs 1.6 months, respectively; *P* < 0.001) [65]. Subgroup analysis identified patients with favourable baseline factors such as higher performance status, normal LDH and reduced disease burden had greater benefit of vemurafenib compared to dacarbazine [66]. While a 4-year landmark analysis confirmed that this correlated to superior median OS in the vemurafenib groups (13.6 vs 9.7 months, respectively; *P* = 0.03), a similar majority of patients in both treatment arms had progressed (76.5 vs 74.4%, respectively) [66].

Similarly, dabrafenib was compared against dacarbazine (BREAK-3) in the 1L setting [67]. This study allowed patients to freely crossover to dabrafenib upon progression on dacarbazine, and thus, PFS was thus the primary endpoint to minimise the confounding effects on OS. ORR was superior in patients receiving first-line dabrafenib compared to dacarbazine (50% vs 6%, respectively) as was PFS (median 6.7 vs 2.9 months), which was consistent across subgroups stratified by performance status, LDH level and staging. However, durable responses were infrequent — with a 5-year PFS rate of only 12% in the dabrafenib arm compared to 0 in the dacarbazine, while the 5-year OS rate was similar between the two treatments (24% and 22%, respectively) [68]. BREAK-3 also provides valuable data on QoL benefits to patients — with a 6-week sub-analysis showing stable or improved QoL in patients receiving dabrafenib versus deterioration in those receiving dacarbazine [69].

Selumetinib is a MEK1/2 inhibitor with similar ORR in melanoma when compared to temozolomide (5.8 vs 9.4%, respectively) in the 1L setting, which was maintained in subgroup analysis of the BRAF-mutant cohort (11.1 vs 10.7%) [70]. Moreover, neither PFS nor OS were significantly extended by selumetinib compared to temozolomide (*P* = 0.70 and 0.10, respectively) — suggesting MEK inhibition is not superior to chemotherapy in unselected cohorts. In contrast, MEK inhibition with binimetinib in patients with NRAS mutations, present in 15–20% of cutaneous melanoma, shows significantly improved ORR when compared to dacarbazine in the 1L/1L + setting (15% vs 9%, respectively; *P* = 0.015) and PFS (2.8 vs 1.5 months, respectively; *P* < 0.001) [71]. Similarly, pimasertib was associated with significantly higher ORR (27% vs 14%, *P* = 0.05) and longer PFS (13 vs 7 months, *P* = 0.002) compared to dacarbazine (1L) [72]. However, neither study demonstrated significantly improved OS by MEK inhibition over chemotherapy (11.0 vs 10.1 months in NEMO, *P* = 0.50; 9 vs 11 months in NCT01693068).

METRIC is another randomised trial of trametinib versus investigators’ choice chemotherapy (dacarbazine or paclitaxel) in BRAF-mutant melanoma (1L/1L +) [73]. Higher ORR was observed in the trametinib versus chemotherapy arms (40% vs 14%, respectively); and, in addition, of the 65% of chemotherapy-treated patients who crossed over to trametinib, 44% then experienced an objective response. This corresponded to improved PFS (4.9 vs 1.5 months, respectively) as well as 5-year survival rates (32% vs 13.3%), even accounting for the high crossover rate. In addition, trametinib was associated with significantly reduced deterioration of QoL compared to chemotherapy over 12 weeks with less functional impairment and symptom burden [74].

## When Can Chemotherapy Still Be Considered and When Should It Not?

All-in-all, given the lack of high-quality evidence supporting the meaningful benefits of chemotherapy in cutaneous melanoma, particularly given their inferiority to contemporary treatments and high toxicity rates, their utility is limited. However, there are a few scenarios in which chemotherapy may still be valuable. These are summarised in Table 3, including the evidence level supporting or discouraging their use (as per the Oxford Centre for Evidence-Based Medicine grading [75]).

**Table 3:** Scenario-based considerations of chemotherapy use in the contemporary management of melanoma, stratified by the level of evidence supporting their use (per the Oxford Centre of Evidence-Based Medicine grading)

Setting	Considerations	Level of evidence (OCEBM)
Locoregional therapy	• No comparisons between ICIs and isolated limb perfusion for ITMs • Considerable toxicities, need to consider individual patient risk	I
Salvage therapy	• May be considered in patients who progress on first-line treatments who are not eligible for trials • Can be considered in patients with severe toxicities to first-line treatments	III
Brain metastases	• Chemotherapy inferior to ICIs, can be considered as salvage therapy • Unclear if chemotherapy is inferior to TT for brain metastases, cross-trial comparison suggests inferiority	III
Combination with immunotherapy and targeted therapy	• Limited data for combining current immunotherapy and kinase inhibitors • Pre-clinical data suggesting synergistic effect of chemotherapy • No evidence adding chemotherapy enhances outcomes to ICIs or TT	III
Bridge to treatment availability	• Can be used to control cancer related symptoms in patients with high tumour burden while awaiting availability • Can be used to preserve performance status and biochemical markers (e.g. LDH) while awaiting clinical trial or drug availability	V

*ICI*, immune checkpoint inhibitor; *ITM*, in-transit metastases; *LDH*, lactate dehydrogenase; *TT*, targeted therapy

### Locoregional Chemotherapy

Approximately 5–10% of patients with melanoma will develop cutaneous in-transit metastases (ITMs) between the primary tumour and the draining nodal basin. Isolated limb perfusion/infusion involve regional administration of high-dose chemotherapy agents such as melphalan (an alkylating agent), with or without TNF-α, with pooled ORR of 60–80%, median OS of approximately 40 months and low rates of systemic toxicity in patients with ITM-limited disease [76, 77]. A phase 1b trial of isolated limb infusion in combination with nivolumab for ITMs is ongoing (NCT03685890). Electrochemotherapy with bleomycin (intravenous/intratumoural) can also be effective, with pooled ORR of 78% [78]. Given the paucity of data on contemporary treatments in patients with ITMs specifically, with one retrospective study demonstrating an ORR of 56% and 5-year OS of 63% with ICIs [79] (acknowledging likely differences between study cohorts), tumour-directed chemotherapeutics may be valuable alternatives to systemic therapy.

### Salvage Therapy

Given that the majority of patients will ultimately progress on first-line treatments [2, 52], there remains a need to consider salvage treatment options for these patients. European Consensus guidelines suggest chemotherapy should only be considered as a last-line option in patients who have progressed on ICIs and (if BRAF-mutant) BRAF/MEKis, if enrolment in a clinical trial is not possible [80]. It has been postulated ICIs may potentiate efficacy of subsequent chemotherapy by priming CD8 + T cell activity, supported by small cohort studies [81]. However, a retrospective multicentre study demonstrated low ORR and PFS of 12.4% and 2.6 months, respectively, in patients treated with chemotherapy following ICI failure [82••]. Taxanes had the highest ORR (25%) and PFS (3.9 months); however, this was not statistically significant on multivariate analysis. This suggested overall low activity of chemotherapy which should not thus be the automatic treatment sequence following ICI/TT failure and emphasised the importance of instead seeking clinical trials or, if not feasible, informed patient-centred decision-making between chemotherapy versus BSC.

Similarly, in patients whom treatment with first-line therapies may be relatively contraindicated (e.g. ICIs in solid organ transplant recipients or those with uncontrolled autoimmune diseases [80]) who are thus also likely to be ineligible for clinical trials, the low clinical activity of chemotherapy should be carefully balanced with the potential harms to QoL when discussing active versus palliative therapy goals with patients. However, no trials specifically evaluated outcomes of chemotherapy in this population, and thus, high-quality evidence supporting or discouraging their use is lacking.

The role of predictive tumour biomarkers in guiding targeted chemotherapy regimens in melanoma remains largely unexplored. Alkylating agents such as dacarbazine, temozolomide and fotemustine induce DNA target lesions such as O^6^-methylguanine and O^6^-chlorethylguanine, which ordinarily can be repaired by O^6^-methylguanine-DNA methyltransferase (MGMT). Deficiency of MGMT may therefore indicate tumours more prone to alkylating chemotherapy, as is routinely screened in glioblastoma [83]. However, there are conflicting data correlating MGMT expression and response to dacarbazine between retrospective cohort analyses [84, 85], and no prospective studies investigate pre-treatment MGMT as a prognostic biomarker for chemotherapy in melanoma. Similarly, in vitro studies suggest mismatch repair protein deficiency (~ 25% of cutaneous melanoma) may predict resistance to alkylating agents in melanoma [86], while homologous recombination defects (~ 20% of cutaneous melanoma) may predict sensitivity to platins [87]. However, these have not been validated with clinical data. Pre-treatment biomarkers are an area in need of further research in melanoma to guide patient counselling and treatment stratification, particularly in the context of salvage therapy.

### Brain Metastases

Most trials directly demonstrating inferiority of chemotherapy to ICIs and TT excluded patients with active brain metastases. Agents such as temozolomide or fotemustine may be considered in these patients given their reported intracranial ORRs of up to 25% [15]. Yet, both anti-CTLA-4- and anti-PD-1-based immunotherapies [88–90], as well as BRAF and MEK inhibitor combinations [91], have demonstrated higher ORRs and longer OS. Thus, chemotherapy should only be reserved for patients with CNS metastases who progress on or do not tolerate first-line ICIs/TT and trials are unavailable and always carefully considered against BSC.

### Combined with Immunotherapy or Targeted Therapy

Prior to the introduction of ICIs, chemotherapy was frequently combined with early immunotherapies including IL-2 and IFN-α, although these combinations were more toxic and did not improve OS [51]. It has been suggested chemotherapy-induced necroptosis of cancer cells may increase immunogenic antigen exposure or upregulation of co-inhibitory ligands such as PD-L1, thereby augmenting ICI efficacy. Chemotherapy also has the potential to increase the ratio of effector to regulatory T cells in the tumour microenvironment to increase the cytotoxic potential of checkpoint inhibition, as observed in ex vivo models of docetaxel-treated breast cancer and cisplatin-treated lung cancer [92]. This corresponds to clinical trials showing greater efficacy of nab-paclitaxel and atezolizumab in triple-negative breast cancer [93] or pembrolizumab and platins in non–small cell lung cancer [94] compared to chemotherapy alone.

A small retrospective series of 60 melanoma patients who progressed on anti-PD-1 showed high activity of subsequent chemotherapy plus PD-1 inhibition (ORR 59% and median OS 3.5 years) [95•]. However, the prospective randomised trial NIBIT-M2 in patients with CNS metastases showed no meaningful benefits of ipilimumab addition to fotemustine chemotherapy, and in turn, both were vastly inferior to ipilimumab plus nivolumab [64••]. Moreover, 5-year survival of ipilimumab plus dacarbazine and ipilimumab alone is comparable (18 vs 26%, respectively) between the CA184-024 [62] and Checkmate-067 [3] trials (both in the 1L setting). While this warrants further research in melanoma, at present, there is limited evidence supporting addition of chemotherapy to ICIs, and these should not be used outside of clinical trials.

Similarly, pre-clinical data suggests chemotherapy plus TT may inhibit ATM-dependent DNA repair to kill melanoma cells, including in BRAF wild-type lines [96]. In vitro data also suggests a possible synergistic effect of sequencing temozolomide after vemurafenib in BRAF-mutant melanoma cells, without cross-resistance [97]. One randomised trial suggests the addition of trametinib to paclitaxel improves ORR (42% vs 13%, *P* = 0.01) and PFS (5.2 vs 3.4 months, *P* = 0.04) but not OS (9.4 vs 10.8 months, *P* = 0.18) compared to paclitaxel monotherapy in BRAF wild-type melanoma, although this was not compared against trametinib alone [98]. Therefore, no evidence supports combined chemotherapy and TT at present beyond clinical trials.

### Bridge to Treatment Availability

Many first-line recommended treatments for melanoma as per contemporary guidelines are associated with significant costs and may not be readily available to every patient. For example, a 2016 availability analysis identified that, other than dacarbazine, almost all lower-income European countries either did not have access to contemporary melanoma treatments or, if they did, patients were subjected to full out-of-pocket costs [99]. Furthermore, a 2020 survey of oncologists in 82 countries suggested only 9–54% of patients in low- to middle-resource countries would have universal availability to critical cancer treatments, particularly ICIs [100]. In such cases, chemotherapy may be considered to reduce melanoma-related symptoms in patients with high tumour burden (particularly regimens with consistent ORRs and manageable AEs such as dacarbazine, fotemustine or nab-paclitaxel) even if survival is not significantly impacted while awaiting access.

Similarly, chemotherapy can be considered as a ‘bridge’ in selected cases while awaiting recruitment in a clinical trial. This can include preserving or reducing the deterioration of performance status which may affect otherwise elibility [101] or preserving biochemical markers such as LDH as baseline prognostic factors upon subsequent treatment with immunotherapy or TT [102].

## Conclusion

Although chemotherapy has been widely studied and used in the historical treatment of cutaneous melanoma, its current utility is limited considering the significant superiority of ICIs and BRAF/MEKis. Chemotherapy agents are associated with low objective responses and unclear survival benefits compared to BSC, while their treatment-related toxicities may compromise patient QoL to a greater degree. Therefore, with a few exceptions such as locoregional tumour-directed chemotherapy, salvage therapy after first-line treatment failure (where clinical trials are not available) and as a bridge to treatment availability, traditional cytotoxic chemotherapy regimens should not be considered as standard-of-care in the contemporary management of metastatic melanoma. Our review has focused on metastatic cutaneous melanoma, and therefore, rarer subtypes such as mucosal or uveal melanoma, with their associated evolving treatment paradigms, are beyond the scope of this report.

## Data Availability

Data sharing not applicable as no new data were generated in this manuscript.
